# Staphylococcal species composition in the skin microbiota of domestic pigeons (*Columba livia domestica*)

**DOI:** 10.1371/journal.pone.0287261

**Published:** 2023-07-12

**Authors:** Ewa Szczuka, Maria Wesołowska, Adrianna Krawiec, Jakub Z. Kosicki

**Affiliations:** 1 Department of Microbiology, Institute of Experimental Biology, Faculty of Biology, Adam Mickiewicz University, Poznań, Poland; 2 Department of Avian Biology and Ecology, Faculty of Biology, Adam Mickiewicz University, Poznań, Poland; Khoo Teck Puat Hospital, SINGAPORE

## Abstract

Staphylococci are a natural component of the skin microbiota of many organisms, including humans and birds. As opportunistic pathogens, they can cause a variety of infections in humans. The close contact between domestic pigeons and their owners provide an opportunity for exchange of skin-associated bacteria. In this study, 41 healthy racing pigeons were tested. Staphylococci were detected on the skin of each bird (41/41, 100%). Isolates were identified at the species level using matrix-assisted laser desorption ionization time of flight mass spectrometry (MALDI-TOF MS). The diversity of the *Staphylococcus* species was relatively high and coagulase-negative staphylococci (CoNS) were predominantly isolated. In total, ten different staphylococcal species were identified. *S*. *lentus* (19/41, 46.3%) was noted most frequently. The pigeon skin was also inhabited by *S*. *xylosus* (6/41, 14.6%), *S*. *equorum* (4/41, 9.8%), *S*. *hyicus* (3/41, 7.3%), *S*. *intermedius* (2/41, 4.9%), *S*. *sciuri* (2/41, 4.9%), *S*. *vitulinus* (2/41, 4.9%), *S*. *lugdunensis* (1/41, 2.4%), *S*. *hominis* (1/41, 2.4%), and *S*. *auricularis* (1/41, 2.4%). Our results indicate that domestic pigeons may carry pathogens with zoonotic potential. All strains were susceptible to 12 antibiotics (ciprofloxacin, clindamycin chloramphenicol, erythromycin, fosfomycin, gentamicin, levofloxacin, norfloxacin, rifampicin, tobramycin, trimethoprim/sulfamethoxazole, vancomycin) representing 8 different classes. None isolate displayed a multidrug-resistant phenotype. Resistance to tetracycline (6/41, 14.6%) and to penicillin (4/41, 9.7%) was shown. The *mec*A gene was not detected in the examined strains and no methicillin-resistant staphylococci were found on the skin of the healthy pigeons.

## Introduction

Staphylococci are widely distributed on human and animal skin, where they constitute the majority of the commensal bacterial microbiota [1]. The coagulase-negative staphylococci (CoNS) *Staphylococcus epidermidis* are the most prevalent bacteria of the human skin microbiota, whereas coagulase-positive staphylococci (CoPS) *S*. *intermedius* and *S*. *pseudintermedius* are most common species in domestic animals such as dogs and cats [2, 3]. Staphylococci are also opportunistic pathogens associated with serious infections of humans such as bacteraemia, endocarditis, prosthetic joint infections, eye and surgical site infections. Besides highly virulent *S*. *aureus*, there are also less pathogenic staphylococci, e.g. *S*. *epidermidis*, *S*. *haemolyticus*, and *S*. *lugdunensis*, and staphylococci classified as non-pathogenic. However, even non-pathogenic staphylococcal species may cause many infections, particularly in critically ill and long-term hospitalised patients [4]. In pigeons (*Columba livia domestica*), most staphylococcal infections are caused by *S*. *aureus*. It is recognised as a causative agent of skin inflammation, wound infections, skin abscesses, conjunctivitis, meningitis, and brain abscesses [5–8]. Particularly, young pigeons exposed to increasing physical demands during the race season and birds with simultaneous or earlier infections with other pathogens are susceptible to staphylococcal infection [9]. As mentioned above, staphylococci constitute part of the normal skin microbiota of birds [4]. *S*. *intermedius* was previously described as a species isolated from the nostrils of pigeons [10–13]. Other studies reported the presence of *S*. *delphini*, *S*. *schleiferi*, and *S*. *aureus* in nasopharynx, cloacae and posterior nares of pigeons [5, 14]. It is worth noting that most studies involving birds have focused on the gut microbiota of economically significant birds (chickens and turkeys) [15].

Methicillin-resistant staphylococci are the most important in human and veterinary medicine. Methicillin resistance is based on the expression of a modified penicillin-binding protein transpeptidase, with a low affinity for beta-lactams, known as PBP2a or PBP2′, which is encoded by the *mec*A gene. Notably, methicillin-resistant staphylococci (MRS) often exhibit multidrug resistance (i.e. to at least three non- beta-lactam antibiotics), which extremely limits therapeutic options [16]. Staphylococci can be transmitted from animal- to- human and from human to animal [13, 17]. For instance, owners of domestic animals suffering from *S*. *pseudintermedius*- infections are more likely to be colonised by these bacteria than persons without contact with colonized or infected dogs or cats [18–20]. Recently, the concept of "One Health", clearly recognized the link between human health and animal health [21].

Microbiome studies have been a focus of interest of researchers for the past few years; however, most studies addressing this issue concern humans and domestic animals: dogs and cats [22]. Birds have received less attention. This study was undertaken to determine the composition of staphylococcal species in the pigeon skin microbiota. Our study also aimed to determine whether the pigeon skin is colonised by methicillin-resistant *Staphylococcus* sp. (MRS).

## Materials and methods

### Sampling and bacterial identification

This study was carried out in strict accordance with the recommendations in the Guide for the National Ethical Committee for Experiments on Animals (KKE). KKE is an independent expert team operating under the Act of 15 January 2015 on the protection of animals used for scientific or educational purposes (Journal of Laws of 2019, item 1392, as amended). KKE performs the function of the national committee for the protection of animals used for scientific purposes, in compliance with the provisions of Directive 2010/63/EU of the European Parliament and of the Council of 22 September 2010 on the protection of animals used for scientific purposes (L 276/33). Taking domestic pigeon skin swabs does not require the consent of the National Ethical Committee for Animal Experiments (KKE). We declare that the pigeons did not suffer. The owners took appropriate care and were emotionally attached to their birds. Samples were collected from the skin of racing pigeons aged from 4 months to two years at the beginning of the race season. In total, forty one pigeons from five different lofts: eight pigeons from each of four lofts and nine birds from one loft were sampled. The lofts were located in Wielkopolska Province in Poland. The pigeons did not show clinical signs of any disease. The birds were included in the examination group after receiving permission from their owners to take samples. The samples were taken by vigorously swabbing skin under the wings i.e. armpit with a sterile cotton swab (Deltalab, Spain) moistened with sterile physiological saline. Each skin swab sample was placed in 2 ml of brain-heart infusion broth (BHI) (Oxoid, United Kingdom) and incubated at 37°C in ambient air for 24 hours. Then, 10μl of this culture was inoculated on Mannitol Salt Agar (Oxoid, United Kingdom). The plates were then incubated for 24 hours. Morphologically distinguishable staphylococcal colonies were picked with a needle and transferred to BHI agar to obtain pure cultures. The preliminary identification of staphylococci based on Gram staining and detection of catalase and coagulase production. Next the bacteria were identified at the species level by matrix-assisted laser desorption ionization time of flight mass spectrometry (MALDI-TOF MS) (Bruker, USA) as previously described [23]. We used the chi-square test to analyze differences between number of bacterial isolates.

### Susceptibility testing

The antimicrobial susceptibility of the staphylococci was tested using the agar disk diffusion method according to the CLSI standards for the following antimicrobial agents (μg/disk): cefoxitin (30), ciprofloxacin (5), clindamycin (2), chloramphenicol (30), erythromycin (15), fosfomycin (5), gentamicin (10), levofloxacin (5), norfloxacin (10), penicillin G (10), rifampicin (5), tetracycline (30), tobramycin (10) and trimethoprim/sulfamethoxazole (1.25 + 23.75). To determine vancomycin resistance, we used vancomycin MIC TEST STRIP (Liofilchem, Roseto degli Abruzzi Italy) [24].

### Preparation of total DNA for PCR and detection of the *mec*A gene

The total DNA was isolated and purified using the Genomic Mini DNA kit (A&A Biotechnology, Gdynia, Poland). The presence of antibiotic resistance gene *mecA*, was assessed using PCR assay. The thermal cycling condition was as follows: initial denaturation at 94°C for 5 min, 30 cycles (denaturation, 94°C 1 min; annealing, 50°C 1 min; extension, 72°C 2 min), final extension at 72°C for 10 min and held at 4°C. The primer set (F GTG AAG ATA TAC CAA GTG ATT and R ATG CGC TAT AGA TTG AAA GGA T) used in this method amplifies a 147bp fragment of the *mec*A gene [25]. Positive control (DNA from *S*. *aureus* ATCC strain 43300) was used to check the reliability of the PCR reactions.

## Results

The research included 41 staphylococcal strains from the skin of racing pigeons. Staphylococci were detected on the skin of each bird (41/41, 100%). *S*. *lentus* was identified in 19 of the 41 samples (46.3%) tested and it was found to be a dominant species. The pigeon skin was also inhabited by: *S*. *xylosus*, *S*. *equorum*, *S*. *hyicus*, *S*. *intermedius*, *S*. *sciuri*, *S*. *vitulinus*, *S*. *lugdunensis*, *S*. *hominis*, and *S*. *auricularis* (Table 1). We found that the number of isolates these species was different (chi-square = 160.86, df = 9, p < 0.001). Our results indicated that the skin of the domestic pigeons was dominated by CoNS (36/41, 87.8%) i.e. *S*. *lentus*, *S*. *xylosus*, *S*. *equorum*, *S*. *sciuri*, *S*. *vitulinus*, *S*. *lugdunensis*, *S*. *hominis* and *S*. *auricularis*. Five out of 41 (12.2%) of CoPS were obtained, three were identified as *S*. *hyicus* and two as *S*. *intermedius*. Noteworthy, there were pigeons carrying different staphylococcal species in each loft. In loft 1, there were pigeons carrying *S*. *luteus*, *S*. *xylosus*, *S*. *equorum*, *S*. *hyicus*, and *S*. *lugdunensis*. The skin of pigeons from loft 2 was colonised by *S*. *luteus*, *S*. *xylosus*, *S*. *equorum*, *S*. *intermedius*, and *S*. *sciuri*, whereas pigeons in loft 3 were colonised by *S*. *luteus*, *S*. *xylosus*, *S*. *sciuri*, and *S*. *vitulinus*. Six different species, i.e. *S*. *luteus*, *S*. *xylosus*, *S*. *equorum*, *S*. *hyicus*, *S*. *hominis*, and *S*. *auricularis* were isolated from pigeons in loft 4. *S*. *luteus*, *S*. *xylosus*, *S*. *equorum*, *S*. *hyicus*, and *S*. *intermedius* were isolated from pigeons originating from loft 5 (S1 Table).

**Table 1:** Distribution of 41 strains of the *Staphylococcus* species in examined pigeons, antibiotic resistance and presence of *mec*A gene.

Species	Number of isolates (%)	Resistance (number of resistant strains)	Presence of *mec*A gene
*S*. *lentus* ^CoNS^	19 (46.3%)	Tetracycline (4) Penicillin (3)	0
*S*. *xylosus* ^CoPS^	6 (14.6%)	-	0
*S*. *equorum* ^CoPS^	4 (9.8%)	-	0
*S*. *hyicus* ^CoPS^	3 (7.3%)	Tetracycline (1)	0
*S*. *intermedius* ^CoPS^	2 (4.9%)	Tetracycline (1)	0
*S*. *sciuri* ^CoPS^	2 (4.9%)	-	0
*S*. *vitulinus* ^CoPS^	2 (4.9%)	-	0
*S*. *lugdunensis* ^CoPS^	1 (2.4%)	-	0
*S*. *hominis* ^CoPS^	1 (2.4%)	Penicillin (1)	0
*S*. *auricularis* ^CoPS^	1 (2.4%)	-	0

All isolates were susceptible to ciprofloxacin, clindamycin, chloramphenicol, erythromycin, fosfomycin, gentamicin, levofloxacin, norfloxacin, rifampicin, tobramycin, trimethoprim/sulfamethoxazole, and vancomycin. Only six and four strains were resistant to tetracycline (6/41, 14.6%) and penicillin (4/41, 9.7%), respectively. Among the six tetracycline-resistant isolates, three different *Staphylococcus* species were identified: *S*. *lentus* (4/41, 9.7%), *S*. *hyicus* (1/41, 2.4%) and *S*. *intermedius* (1/41, 2.4%). The penicillin-resistant isolates represented the species *S*. *lentus* (3/41, 7.3%) and *S*. *hominis* (1/41, 2.4%). None of the isolates displayed a multidrug-resistant (MDR) phenotype. MDR was defined as acquired non-susceptibility to at least one agent in three or more antimicrobial classes [26]. Moreover, the *mec*A gene was not detected in the examined staphylococci and no methicillin-resistant staphylococci were identified.

## Discussion

The domestic (racing) pigeons included in the present study came from zones close to humans; therefore there was a strong likelihood of contact between the pigeons and their owner. It is well known that bacteria associated with the skin of animal can be transmitted to people via direct contact [27]. The objective of this study was to investigate the composition of staphylococcal species in healthy pigeons. In total, ten different staphylococcal species were identified: *S*. *lentus*, *S*. *xylosus*, *S*. *equorum*, *S*. *hyicus*, *S*. *intermedius*, *S*. *sciuri*, *S*. *vitulinus*, *S*. *lugdunensis*, *S*. *hominis* and *S*. *auricularis*. Importantly, the phylogenomic analyses of the *Staphylococcaceae* family suggest the taxonomic reassignment of five *Staphylococcus* species i.e. *S*. *sciuri*, *S*. *fleurettii*, *S*. *lentus*, *S*. *stepanovicii* and *S*. *vitulinus* to the novel genus *Mammaliicoccus* with *Mammaliicoccus sciuri* as the type species [28]. In our study *S*. *lentus* (proposed to be reclassified as *Mammaliicoccus lentus*) was a dominant species. Moreover, *S*. *lentus* was found in the skin of pigeons from each loft. The diversity of *Staphylococcus* species was high in each loft. We found isolates of four to six recognised *Staphylococcus* species. In our study, the spectrum of the staphylococcal species (i.e. eight recognized species of CoNS and two of CoPS) in the healthy domestic pigeons was wider than that found in pigeons living in Kobe, Japan [29]. Noteworthy, 38 pigeons were sampled and 20 (52.6%) Staphylococcal strains were isolated. The skin of these pigeons was dominated by *S*. *xylosus*, in contrast to our studies, where *S*. *lentus* was usually isolated from the pigeons skin. In Turkish research, *S*. *delphini* was isolated from the nasopharynx of healthy domestic pigeons [5]. According to Ross et al. [30] the geographic location and living environments may influence the skin microbiome. Moreover, the moulting season or the race season may affect the presence of commensal bacteria in pigeons [9, 31]. Little is known about the prevalence of staphylococci in pigeons from European countries. Reports from Slovakia indicated that the oropharynx of healthy pigeons was colonised by *S*. *intermedius*, *S*. *schleiferi*, *S*. *epidermidis*, *S*. *xylosus*, *S*. *simulans*, *S warneri*, and *S*. *aureus* [9]. Other studies focused on coagulase-positive staphylococci, reported the presence of *S*. *delphini*, *S*. *intermedius*, *S*. *schleiferi* and *S*. *aureus* in cloacae and posterior nares of pigeons [14]. As show by the results of our studies, the skin of pigeons was colonised by *S*. *intermedius* but not by *S*. *delphini*, *S*. *schleiferi* or *S*. *aureus*.

The knowledge of the pigeons skin microbiota is necessary for estimation of the risk of colonization and infections of humans by microorganisms with zoonotic potential. The pigeon owners assured us that they did not suffer from staphylococcal infections. Therefore we did not examine the samples from the pigeon owners. In this context, it is interesting to note, that the skin of the pigeons was not colonized by *S*. *aureus*. In birds, most staphylococcal infections are caused by *S*. *aureus*. Recently Chrobak-Chmiel et al. [7] has reported that *S*. *aureus* were isolated from the oral cavity of a racing pigeon with yellowish nodular lesions on the eyelids, as well as protuberant black pocks in the nostrils, cere region, and lower beak. Of note, companion animals in general are often in close contact with their owners, therefore risk of bacterial transfer between these animals and humans and vice versa has to be taken into consideration. Steinagel et al. [32] reported a case of endocarditis developing in the Umbrella Cockatoo. The translocation from the owner’s skin from the repeated manual manipulation may have been a source of the *S*. *aureus* infections in this cockatoo. Similarly, the methicillin-resistant *S*. *aureus*–associated dermatitis in a Congo African Grey Parrot (*Psittacus erithacus erithacus*) may have been associated with the acquisition of *S*. *aureus* from a parrot owner [33]. Until now, no human-to-pigeon disease transmission has been reported. In this study, the pigeons was colonised by *S*. *lentus*. Previous reports, have demonstrated that *S*. *lentus* is a commensal bacterium colonizing the skin of several animal species, including pets, farm animals and wild animals. Interestingly, people working with animals have also been reported to be carriers of *S*. *lentus*. This bacterium can cause several serious human infections, including sinusitis, endocarditis, peritonitis, septic shock, urinary tract infection and wound infections [34–36]. Other CoNS species, such as *S*. *xylosus* and *S*. *equorum* have been found in the present study in the pigeon skin microbiota in lower number of pigeons. *S*. *xylosus* is defined as a nonpathogenic *Staphylococcus*, but some *S*. *xylosus* strains can occasionally cause human infections [37]. For example, Dordet-Frison et al. [38] reported a case of erythema nodosum that developed in a young woman with *S*. *xylosus* septicaemia. Similarly, *S*. *equorum* has rarely been reported as a pathogen in humans [39]. This species has been described as an etiological agent of urinary tract infections, endocarditis, pyelonephritis, or pneumonia. We also isolated members of the *S*. *sciuri* group (proposed to be reclassified as *Mammaliicoccus* group), i.e. *S*. *sciuri*, *S*. *vitulinus* and *S*. *lentus* mentioned above from skin of the pigeons. Although these organisms are part of the microbiota of many animals, they appeared to be implicated in the aetiology of a variety of human infections [40]. Moreover, it has been reported that some pathogenic strains of *S*. *sciuri* caused fatal exudative epidermitis in piglets. These findings prioritise *S*. *sciuri* as zoonotic agents that may be of importance to human medicine [41]. The skin of the pigeons was colonized by coagulase-positive staphylococci: *S*. *intermedius* and *S*. *hyicus*. Of note, *S*. *intermedius* strains are not pathogenic for the pigeons, they might cause infections in other animals, particularly in dogs [42]. *S*. *intermedius* can be involved in soft tissue wound infections in humans, particularly in dog bite wounds, and more rarely in endocarditis, pneumonia, and brain abscesses [43]. Similarly, infections caused by *S*. *hyicus* are usually rare. *S*. *lugdunensis* is a *Staphylococcus* species that deserves attention, as its virulence is higher than that of other CoNS [1]. This bacterium is responsible for deep soft tissue infections in humans. This study suggests, that pigeons may be a source of staphylococcal infections for their owner.

It has previously been suggested that birds can carry antibiotic-resistant bacteria [44, 45]. The present study indicated a low level of resistance to antibiotics among staphylococci isolated from pigeons. All strains were susceptible to 12 antibiotics representing 8 different classes (i.e. aminoglycosides, fluoroquinolones, glycopeptides, macrolides, lincosamides, rifampin, trimethoprim/sulfamethoxazole, fosfomycin). Only six and four strains were resistant to tetracycline (14.6%) and penicillin (9.7%), respectively. The rates of tetracycline resistance in this study are comparable to the rates of 9.7% previously observed in *S*. *intermedius* isolated from pigeons in Japan [46]. Significantly higher occurrence of resistance to antibiotics (37.3%) was observed in *S*. *aureus* strains isolated from a pigeon slaughterhouse in Italy [47]. Approximately, 68% of the *S*. *intermedius* strains isolated in Germany exhibited resistance to tetracycline [42]. In contrast, all *S*. *aureus* strains isolated from faeces of urban pigeons in Brazil were susceptible to all tested antibiotics [48]. Also, all staphylococci isolated from healthy pigeons in the Czech Republic were sensitive to the nine tested antibiotics [10]. In the present study, no colonisation of pigeon skin by multidrug-resistant bacteria was detected. Also, the *mec*A gene responsible for methicillin resistance was not detected in the examined staphylococci. In contrast, Stenzel et al. [49] reported MRS strains isolated from pigeons originating from flocks located all over Poland. Also, a methicillin-resistant *S*. *aureus* strain was isolated from the oral cavity of a racing pigeon with lesions typical for pigeon pox virus infection [7]. MRSA were present in the air in pigeons exhibitions in Denmark in January 2020 [50]. It is interesting to note that the prevalence of methicillin resistance is greater among staphylococci from dogs and cats, and this observation probably partly relies on the frequent antibiotic administration and the close contact dogs and cats with humans.

## Conclusions

The concept of "One Health", clearly recognized the link between human health and animal health. Our results indicate that, the pigeon skin microbiota was inhabited by: *S*. *lentus S*. *xylosus*, *S*. *equorum*, *S*. *hyicus*, *S*. *intermedius*, *S*. *sciuri*, *S*. *vitulinus*, *S*. *lugdunensis*, *S*. *hominis*, and *S*. *auricularis*. These staphylococcal species are opportunistic pathogens associated with serious human infections. We think, that the close contact between domestic pigeons and their owners provide an opportunity for exchange of skin-associated bacteria.

## Supporting information

S1 Table Prevalence (%) of staphylococci strains isolated from pigeons from different lofts. (PDF)
