# Supplementary material for: Staphylococcal species composition in the skin microbiota of domestic pigeons (Columba livia domestica)
Source: PLoS One. 2023 Jul 12;18(7):e0287261. doi: 10.1371/journal.pone.0287261 (PMC10337865; doi:10.1371/journal.pone.0287261)
Supplement: S1 Table — (PDF) [file pone.0287261.s001.pdf]

**S1 Table.**

**Prevalence (%) of staphylococci isolated from pigeons from different lofts.**

| Loft | Number of pigeons | Species (number of strains ) |
| --- | --- | --- |
| 1 | 8 (19.5%) | *S. luteus* (4)<br>*S. xylosus* (1)<br>*S. equorum* (1)<br>*S. hyicus* (1)<br>*S. lugdunensis* (1) |
| 2 | 8 (19.5%) | *S. luteus* (4)<br>*S. xylosus* (1)<br>*S. equorum* (1)<br>*S. intermedius* (1)<br>*S. sciuri* (1) |
| 3 | 8 (19.5%) | *S. luteus* (3)<br>*S. xylosus* (2)<br>*S. sciuri* (1)<br>*S. vitulinus* (2) |
| 4 | 8 (19.5%) | *S. luteus* (3)<br>*S. xylosus* (1)<br>*S. equorum* (1)<br>*S. hyicus* (1)<br>*S. hominis* (1)<br>*S. auricularis* (1) |
| 5 | 9 (22%) | *S. luteus* (5)<br>*S. xylosus* (1 )<br>*S. equorum* (1)<br>*S. hyicus* (1)<br>*S. intermedius* (1) |
